# Effect of biosecurity‐based interventions on broiler crude mortality rate at an early stage of production in the small‐scale farming system in Bangladesh

**DOI:** 10.1002/vms3.1230

**Published:** 2023-07-27

**Authors:** Md Omar Faroque, Md Rasel Prank, Md Ahaduzzaman

**Affiliations:** ^1^ Department of Medicine and Surgery Chattogram Veterinary and Animal Sciences University (CVASU) Chittagong Bangladesh

**Keywords:** Bangladesh, biosecurity, broiler, mortality, small‐scale farm

## Abstract

**Background:**

Biosecurity‐based interventions are important health strategies for the control of infectious disease and improve productivity in broiler. There are various reasons why broilers die at the early stages of production; however, biosecurity measures are crucial in reducing the risk of disease prevalence and can therefore affect the overall deaths in a flock for a specific period (crude mortality rate [CMR]).

**Objectives:**

This study was designed to determine the current biosecurity situation on small‐scale broiler farms in Bangladesh and its relationship to the CMR during the early stages of production.

**Methods:**

A cross‐sectional observational study was conducted on small‐scale broiler farms (*N* = 57) located in peri‐urban areas of Chattogram and Pabna districts of Bangladesh. Descriptive and predictive statistical analyses were performed to estimate the frequency of categorical variables and their effect on CMR.

**Results:**

The findings indicated that around 80% of small‐scale broiler farms lacked adequate biosecurity measures. Both on day 1 (*p* = 0.012) and on days 2–14 (*p* = 0.003), flocks with inadequate biosecurity had considerably higher median CMRs. Farms that were near the neighbourhood used wood shavings as bedding, offered tube well/shallow well or supply line water, maintained a brief empty period (≤5 days) before introducing new flocks and began using antibiotics on day 1 all had significantly higher CMR at both days 1 and 2–14.

**Conclusions:**

The findings point to the necessity of implementing stringent biosecurity measures in broiler production in locations where there is a shortage, which can lower the burden of arbitrary antibiotic usage and will lower total production costs due to crude mortality and management‐related issues.

## Graphical abstract

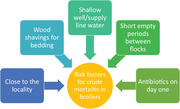

## 1 INTRODUCTION

The practice of biosecurity is a series of essential fundamental precautions or initiatives used to reduce the possibility of the introduction and spread of disease‐causing agents. Farmers need to change their attitudes and become more conscious of risk reduction in all production chain operations for the initiative to be implemented successfully (Dorea et al., 2010; Food and Agriculture Organization of the United Nations (FAO), 2008). Appropriate control of disease‐causing agents through biosecurity largely depends on several factors, such as pathogenesis and mechanism of disease spread and their associated risk factors, farming system and farm location and economic status of the farmers (Conan et al., 2012; Youssef et al., 2021). Careful analysis of these factors can suggest how biosecurity should be implemented, such as whether the focus should be on ‘bio‐exclusion’ that keeps disease agents out or ‘bio‐contaminant’ that keeps disease agents in, or whether it should be on both (FAO, 2008; Delpont et al., 2021). Generally, in broiler farms with good biosecurity practices, a variety of activities, such as immunization, the use of disinfectants, foot baths, personal hygiene, maintaining water quality, limiting visitors numbers, rodent control, proper disposal of dead birds, fumigation and maintaining empty period, are routinely carried out to protect the birds from known and unknown disease hazards responsible for birds’ mortality (Gelaude et al., 2014). These practices can be further categorized into three categories: (i) conceptual biosecurity, the primary level of biosecurity, such as farms should not be located close to populated areas, limiting access by personnel and vehicles and controlling the movement of other animals; (ii) structural biosecurity, the secondary level of biosecurity which focuses on physical factors like drainage, air filtration systems and housing design; and (iii) operational biosecurity, the tertiary level of biosecurity, and it deals with procedures and actions that are routinely assessed as part of a disease management strategy in response to an emergency (Torremorell, 2021). However, in the small‐scale broiler production system in the developing world, farmers are reluctant to adopt biosecurity measures due to financial and knowledge constraints (FAO, 2008; Rimi et al., 2017).

Bangladesh is one of the most densely populated countries in the world where the majority of people live in peri‐urban and rural areas. Bangladesh's per capita income was 1044 USD in 2013 and is now expected to reach 2226 USD in 2021 (World Bank, 2021). As Bangladesh farmers’ economic situation continually improves, there is a growing need for better biosecurity measures to boost productivity. In Bangladesh, poultry, especially broiler chicken, is a substantial contributor to the national economy and is a cost‐effective source of high‐quality protein (Kamruzzaman et al., 2021). The broiler is produced in Bangladesh mostly in the small‐scale production system (flock size of approximately 1000 birds) by individual farmers in peri‐urban regions and to a less extent in large‐scale commercial production systems by companies in dedicated rural areas (Kawsar et al., 2013). Small‐scale broiler farming in Bangladesh is becoming increasingly competitive and frequently experiences tumultuous times when producers must split profits or losses due to bird death and production costs associated with therapeutics caused by disease outbreaks mostly at the early stage of production possibly due to poor biosecurity measures. There are several studies on the socio‐economic status of broiler farmers, cost–benefit analysis of broiler farming and prevalence studies on disease‐related problems available (Mozumdar et al., 2009; Alam et al., 2014). However, information on biosecurity measures and mortality rates in broilers is limited. The study was therefore designed to test the hypotheses that (1) most small‐scale farms will have poor biosecurity and (2) farms with poor biosecurity will have a higher crude mortality rate (CMR) for broilers.

## 2 MATERIALS AND METHODS

### 2.1 Study area and data collection

A cross‐sectional observational study was conducted to collect data associated with small‐scale (≤1000 birds per flock) broiler farming. Data were collected through direct observation and interviews made with farmers using a structured questionnaire from 57 broiler farms located in peri‐urban areas of Chattogram (*n* = 23) and Pabna districts (*n* = 34). The questionnaire aimed to describe the biosecurity situation in small‐scale broiler farms in Bangladesh to determine whether a preventive measure is being used or whether a given scenario is present or absent. The questions were separated into two main categories, conceptual and structural biosecurity, and emphasis was given to conceptual biosecurity comprised of 18 variables and 3 variables, respectively, on different biosecurity measures. The fewer questions in structural biosecurity were due to less variance in farm structure across Bangladesh peri‐urban areas. In each farm, the visits lasted about 1 h and involved checking farm records (where available), making direct observations of the farm premises (e.g. the distance from locality) and directly interviewing the farm owner (e.g. fate of dead birds). Two veterinarians were present throughout each farm audit. This study period was between February 2021 and April 2021.

### 2.2 Data analysis

Data were recorded in Microsoft Excel and uploaded to JMP14 for analysis. Descriptive statistics were conducted to estimate the frequency (%) of categorical variables. The age‐specific CMR was calculated as the number of deaths in a given period divided by the population exposed to the risk of death in that period and presented as per thousand birds (Centers for Disease Control and Prevention (CDC), 2012). The association between CMR and independent variables was estimated using a non‐parametric Wilcoxon test due to the non‐parametric distribution of data. Results were presented as a median with 25%–75% quartiles and score mean. The non‐parametric regression model was performed to fit continuous variables with the CMR (Li et al., 2012). A *p*‐value ≤0.05 was considered statistically significant.

## 3 RESULTS

### 3.1 Biosecurity status in small‐scale broiler farms

The results are presented in Table 1. A comparatively higher proportion (17/57, 29.8%) of Bangladesh's small‐scale broiler farms were located between 10 and 100 m from the locality. The majority of the farmers (31/57, 54.4%) had 2–5 years of farming experience and had never received any formal training from any organizations (33/57, 57.9%). All of the farms under study were constructed structurally with a netted side wall, hardwood or bamboo floor and a tine shed roof. Both natural and artificial ventilation were used in all farms. Most farms utilized rice husk as bedding (40/57, 70.2%), polyester cloth as a curtain (27/57, 47.4%) and the majority of them received their water supply from shallow wells or tube wells (27/57, 47.4%). Most of the farms (33/57, 57.9%) did not allow visitors. A lack of specific boots for the shed (56/57, 98.3%), regular disinfection (51/57, 89.5%), hand washing (51/57, 89.5%), foot baths (36/57, 67.9%) at the farm entrance and rodent control (54/57, 94.7%) were also noted. The majority of farmers (28/57, 49.1%) provided a 10‐day empty interval between flocks (28/57, 49.1%) and started antibiotic use at day 1 (33/57, 57.9%). Most dead birds were buried (26/57, 45.6%). Every farmer included in this study said that they purchased Grade ‘A’ chicks vaccinated against ND + IB at either day 0 or day 5 and fed their birds using the readily accessible commercial feed. None of the farms had clothing specifically for use within the shed.

**TABLE 1:** Different aspects of biosecurity assessment and their impact on the crude mortality rate per 1000 birds (CMR) at small‐scale broiler farms (*N* = 57) in peri‐urban regions of Chattogram and Pabna district, Bangladesh, during the early phases of production.

			CMR (day 1/1000 birds)			CMR (days 2–14/1000)		
Biosecurity measures	Traits	Frequency (%)	Median (25–75 quantiles	Score mean	*p*‐Value	Median (25–75 quantiles	Score mean	*p*‐Value
Age of farm and farming experience (year)	<2	6 (10.5)	2.0 (0.0–24.0)	27.3	0.14	4.0 (0.9–7.2)	30.3	0.07
	2–5	31 (54.4)	3.0 (0.5–24.7)	31.1		5.0 (1.0–7.3)	31.7	
	>5–10	9 (15.8)	8.5 (0.5–42.5)	34.8		3.9 (0.8–12.5)	33.3	
	>10	11 (19.3)	0.3 (0–40.76)	19.2		1.0 (0.5–1.9)	17.1	
Antibiotic to day‐old chicks	Yes	33 (57.9)	18.8 (1.5–33.5)	37.1	<0.0001	4.0 (1.3–7.7)	32.7	0.04
	No	24 (42.1)	0.4 (0–3.2)	17.9		1.4 (0.5–11.8)	23.9	
Curtain type^a^	Cotton	18 (31.6)	2.0 (0.6–19.3)	29.4	0.98	3.5 (1.2–6.8)	30.8	0.74
	Polyester/tarpaulin	27 (47.4)	3.0 (0.2–24.7)	29.2		1.9 (0.7–6.0)	27.2	
	Polythene	12 (21.0)	1.5 (0.1–25.8)	28.1		2.9 (1.0–7.5)	30.3	
Dedicated boot for shed	Yes	1 (1.7)	2.0 (0.3–19.8)	28.9	0.71	0.0 (0.0–0.0)	3.00	0.11
	No	56 (98.3)	4.0 (4.0–4.0)	35.0		2.8 (1.0–6.5)	29.5	
Dedicated cloth for shed	Yes	0 (0.0)	–	–	–	–	–	–
	No	57 (100.0)	2.0 (0.3–19.5)	29.0		2.5 (1.0–6.3)	29.0	
Disposal of dead birds	Buried	26 (45.6)	1.3 (0.3–18.8)	27.3	0.36	1.7 (0.5–4.3)	24.0	0.08
	City corporation dust bin	4 (7.0)	11.5 (1.7–25.6)	36.9		1.7 (0.2–6.6)	23.3	
	Drain	2 (3.5)	28.0 (25.0–31.0)	47.5		6.3 (6.0–6.7)	43.0	
	Open ground	23 (40.4)	2.0 (0.0–20.0)	28.8		5.0 (1.4–12.0)	35.3	
	Landfill/pit	2 (3.5)	1.5 (0.0–3.0)	19.3		1.3 (1.0–1.5)	19.3	
Distance from locality (m)	<5	15 (26.3)	28.9 (9.0–39.0)	45.9	<0.0001	6.0 (2.0–12.0)	37.6	0.0009
	10–100	17 (29.8)	3.0 (0.0–18.9)	28.6		5.0 (1.9–10.8)	36.2	
	101–200	13 (22.8)	0.7 (0.1–4.3)	22.3		1.0 (0.4–3.5)	18.9	
	>200	12 (21.1)	0.4 (0.0–0.7)	15.8		1.6 (0.4–1.9)	19.0	
Empty period (days)	5	13 (22.8)	36.0 (18.9–67.5)	46.9	<0.0001	7.3 (3.8–12.1)	41.2	0.01
	7	13 (22.8)	0.5 (0.2–1.6)	18.5		1.4 (0.6–1.9)	18.9	
	10	28 (49.1)	2.0 (0.0–9.8)	26.1		3.4 (0.8–5.9)	27.9	
	15	3 (5.3)	3.3 (0.0–3.8)	24.5		5.0 (0.0–11.7)	30.0	
Fate of used litter	Buried	8 (14.1)	9.5 (0.1–27.8)	28.9	0.56	4.5 (1.7–7.4)	34.4	0.69
	Fertilizer	21 (36.8)	2.0 (0.0–14.0)	25.7		2.5 (0.8–7.6)	29.7	
	Fish meal	9 (15.8)	8.5 (1.5–33.8)	34.9		3.0 (0.8–6.7)	28.6	
	Open ground	19 (33.3)	2.0 (0.5–20.0)	29.9		1.4 (0.7–6.0)	26.1	
Feed source	Self‐made	0 (0.0)	–	–	–	–	–	–
	Commercial	57 (100.0)	2.0 (0.3–19.5)	29.0		2.5 (1.0–6.3)	29.0	
Foot bath^a^	Yes	17 (32.1)	0.6 (0.0–2.8)	23.1	<0.0001	1.8 (0.8–7.1)	28.0	0.47
	No	36 (67.9)	19.0 (8.8–33.8)	43.0		5.0 (1.5–6.0)	31.4	
Fumigation after the end of the batch	Yes	57 (100.0)	2.0 (0.3–19.5)	29.0	–	2.5 (1.0–6.3)	29.0	–
	No	0 (0.0)	–	–		–	–	
Hand wash/soap	Yes	6 (10.5)	2.0 (0.2–19.0)	27.5	0.04	2.0 (1.0–7.3)	26.3	0.68
	No	51 (89.5)	14.5 (7.3–31.0)	42.1		3.5 (0.4–5.5)	29.3	
Litter materials	Rice husk	40 (70.2)	0.7 (0.0–2.8)	22.4	<0.0001	1.6 (0.7–4.0)	24.3	0.0045
	Wood shavings	15 (26.3)	25.0 (6.7–60.0)	43.6		6.7 (5.0–12.2)	40.3	
	The mixture of rice husk and wood shavings	2 (3.5)	44.5 (39.0–50.0)	52.5		5.0 (4.0–6.0)	38.0	
Maintain farm record	Yes	45 (78.9)	0.4 (0.0–2.0)	18.8	0.02	2.5 (0.8–6.0)	27.9	0.34
	No	12 (21.1)	3.3 (0.4–26.5)	31.7		3.5 (1.7–10.8)	33.1	
Presence of rodent (rat/mouse)	Yes	54 (94.7)	18.8 (8.5–19.0)	40.5	0.22	6.0 (0.5–8.1)	33.3	0.64
	No	3 (5.3)	2.0 (0.3–21.2)	28.4		2.3 (1.0–6.2)	28.8	
Routine disinfection	Yes	6 (10.5)	2.0 (0.2–19.0)	27.4	0.03	1.9 (1.0–6.7)	28.5	0.52
	No	51 (89.5)	18.9 (8.5–37.3)	42.9		5.2 (2.3–6.5)	33.2	
Training received	DLS	3 (5.3)	28.0 (19.0–28.9)	45.8	0.16	3.9 (1.3–8.7)	47.7	0.01
	NGO	16 (28.1)	2.0 (0.2–16.3)	27.8		1.2 (0.3–4.8)	21.7	
	Both DLS and NGO	5 (8.7)	8.5 (2.4–30.0)	37.6		1.0 (0.3–3.5)	18.2	
	No training	33 (57.9)	1.3 (0.0–19.5)	26.8		8.0 (6.0–12.2)	32.5	
Ventilation system^a^	Both natural and artificial (fan)	57 (100.0)	2.0 (0.3–19.5)	29.0	–	2.5 (0.97–6.33)	29.0	–
Visitors allowed (free access)	Yes	24 (42.1)	18.8 (0.8–33.5)	37.0	<0.0001	3.0 (1.0–7.0)	31.3	0.23
	No	33 (57.9)	0.3 (0.0–2.0)	18.0		1.6 (0.5–5.0)	25.9	
Water source	Deep well	15 (26.3)	0.3 (0.0–0.6)	16.1	0.0003	0.7 (0.3–1.7)	14.3	0.0002
	Tube well	27 (47.4)	18.8 (2.0–31.0)	27.1		4.0 (1.0–7.3)	37.3	
	Supply line	15 (26.3)	2.0 (0.0–6.7)	37.2		5.0 (3.9–11.7)	32.6	

*Note*: –: not estimated.

^a^ Parameters related to structural biosecurity.

### 3.2 Crude mortality rate (CMR) in broilers in small‐scale broiler farms

The median CMR was 2 (0.3–19.5) per 1000 birds at day 1 and 2.5 (1.0–6.3) between day 2 and day 14. According to a bivariate fit, there was a significant positive correlation between CMR at days 1 and 2–14 (CMR at day 2–14 = 2.9 + 0.1 × CMR at day 1; *p* < 0.0001). During the early phases of production, many biosecurity measures significantly influenced CMR in broiler flocks (Table 1). A higher CMR at both days 1 and 2–14 was found in farms that were close to the locality, used wood shavings as bedding materials, provided tube well/shallow well or supply line water, maintained a short empty period (5 days) before introducing new flocks and started using antibiotics from day 1. Additionally, farms that did not use disinfectants or keep farm records, did not have a facility for foot baths or hand washing and allowed visitors also had considerably higher CMR at day 1, indicating improved biosecurity practices might reduce CMR.

## 4 DISCUSSION

In this pilot study, the biosecurity status of small‐scale broiler farms in peri‐urban areas of Bangladesh was investigated. Selected biosecurity metrics were used to relate the biosecurity status to the CMRs at the early stages of production. By actively surveying 57 farms in two areas of Bangladesh, the study's objectives were achieved.

The results of the study completely supported the first hypothesis that most small‐scale farms will have poor biosecurity. Small‐scale broilers farmers in resource‐constrained countries, such as Bangladesh, typically have little to no knowledge of biosecurity concepts because most of them have little formal education and have received little to no training in scientific broiler management due to lack of opportunity and occasionally due to unawareness (Rimi et al., 2017). According to this study, a large percentage of farmers did not obtain training from any government agency or non‐governmental group, and the reason for this was a lack of opportunity. Several studies evaluating biosecurity and risk rationalities linked to avian influenza outbreaks in Bangladesh have found several additional factors that are linked to unsatisfactory biosecurity practices in broiler farms (Rimi et al., 2017; Høg et al., 2019). Those are farmer economic hardships, a lack of available farm space, group behaviour and the limitations of conceptual biosecurity or some farmers who do not see any risk of poor biosecurity. Bangladesh's small‐scale broiler farm biosecurity predicament is comparable to that of other countries like Cameroon (Kouam et al., 2018), Egypt (Eltholth et al., 2016), Ethiopia (Ismael et al., 2021), India (Surendra et al., 2022), Nepal (Dhakal & Gompo, 2022) and Sudan (Mahmoud et al., 2014). In comparison to Bangladesh, the Philippines, a near neighbouring country, has better biosecurity conditions for broiler farms, which is in line with European standards (Tanquilut et al., 2020).

The second hypothesis that farms with poor biosecurity will have a higher CMR for broilers was partially supported by the findings of this study. In farms with poor biosecurity, a higher CMR at the beginning of production was noticed and significantly correlated with numerous biosecurity parameters. As field diagnosis of disease in small‐scale poultry is commonly tentative, and in many cases, farmers do not go for any diagnosis but rather start arbitrary antibiotic use from day 1 to throughout the early production period, this study, therefore, focused on CMR rather than mortality caused by any specific disease. Several studies reported that the occurrence of common infections and/or management‐related problems has been linked to mortality in broilers during the early phases of production (Ali & Hossain, 2010; Islam et al., 2004; Rahman & Samad, 2003). Although basic biosecurity measures involve some additional effort, it is still crucial to adopt them in small‐scale broilers since practising so could reduce the risk of disease outbreaks by 1.5–8.5 times (Fasina et al., 2012). The majority of farmers in this study began adding antibiotics to drinking water regularly as a preventive measure, which not only increases the cost of production overall but also increases the likelihood of the establishment of diseases with multiple drug resistance in the ecology. High CMR in flocks where antimicrobials were used in day‐old chicks is possibly due to the occurrence of diseases or poor immunity due to the destruction of beneficial gut microbiota. A high CMR in flocks that were located in close proximity to the locality used wood shavings as bedding materials, supplied unwholesome water and maintained very short empty periods seems rational, as those parameters could influence the likelihood of the occurrence of several diseases (Castañeda‐Gulla et al., 2020; Julian & Goryo, 1990).

## 5 CONCLUSION

This study showed a primary overview of biosecurity management and its relation to the CMRs among small‐scale broiler farming in Bangladesh. Overall, the structural and conceptual biosecurity status of small‐scale broiler farms was inadequate. The CMR was found to be high in flocks with inadequate biosecurity. The government currently lacks standardized guidelines and monitoring capabilities for the application of biosecurity standards in Bangladesh's small‐scale broiler farms. The current situation would be improved, however, by increasing farmers’ knowledge and consideration of some factors, such as maintaining a suitable farm distance from the neighbourhood, using appropriate bedding materials, providing safe water, maintaining a sufficient empty period between flocks and using antimicrobials judiciously, along with routine regulatory authority monitoring.

## AUTHOR CONTRIBUTIONS

*Data curation; investigation; methodology*: Md Omar Faroque. *Data curation; investigation*: Md Rasel Prank. *Conceptualization; data curation; formal analysis; methodology; software; supervision; writing – original draft*: Md Ahaduzzaman.

## CONFLICT OF INTEREST STATEMENT

The authors declare no conflicts of interest.

## FUNDING INFORMATION

No funding was received.

## ETHICS STATEMENT

No approval from research ethics committees was required to accomplish the goals of this study because it was conducted with routine data from farms.

### PEER REVIEW

The peer review history for this article is available at https://www.webofscience.com/api/gateway/wos/peer‐review/10.1002/vms3.1230.

## Data Availability

No.
